# The Relations of Cancer to Chronic Inflammation

**Published:** 1909-07-03

**Authors:** 


### The Hospital

A Journal of

The Medical Sciences and Hospital Administration.

New Series. No. 123, Vol. V. [No. 1195, Vol. XLVI.] SATURDAY, JULY 3, 1909.

### The Relations of Cancer to Chronic Inflammation.

Although we are still ignorant of the factors which determine the assumption of malignancy by epithelial cells, it has long been known that chronic inflammations are common precursors of cancer. The association of cancer with gall-stones, gastric ulcer, and leucoplakia of the tongue are familiar examples, while nothing can be more conclusive in this respect than the so-called "kangri cancer" of the abdominal wall alluded to in the last report of the Cancer Research Fund. It will be remembered that the natives of Kashmir are in the habit of carrying suspended round the waist a small earthenware vessel called a kangri. This vessel contains a charcoal fire, and is used for the sake of warmth. Its consequences are repeated burns upon the anterior wall of the belly, and a chronic irritation of the skin, upon which squamous carcinoma is frequently implanted, although carcinoma in this situation is a rarity in other parts of the world. It would be easy to multiply examples testifying in the same direction; but this is unnecessary, for the fact that some relation does exist between chronic inflammation and cancer must be accepted as past all question. The nature of the relation is the subject of an excellent and thorough communication by Dr. Victor Bonney appearing in the Seventh Report from the Cancer Research Laboratories of the Middlesex Hospital.

It is an invariable thing to find a cellular infiltration of the connective tissues adjacent to areas of primary carcinoma, but opinion has been divided as to its rôle. One school holds that the inflammatory exudate is entirely secondary and evoked by the irritant action of the proliferating carcinoma cells: the other, that the phase of chronic inflammation paves the way for the malignant proliferation, exercising some definite though unexplained influence over the assumption of malignant characters by the epithelial cells in the neighbourhood. Dr. Bonney's researches, it may be said at once, seem to show conclusively that the inflammatory phase is the precursor and not the successor of the malignant. He finds that in admittedly precarcinomatous conditions such as leucoplakia upon the tongue or vulva, the microscopical picture of the sub-epithelial tissues is identical with that of the connective tissue which surrounds the early down-growths of primary carcinoma. It is characterised by an infiltration of the sub-epithelial connective tissue by lymphocytes and plasma-cells in abundance, the infiltration being attended by a destruction of the normal elastic tissue of this situation. These two features appear to be constant and are beautifully illustrated by a number of plates. Into the de-elasticised and cellular layer descend the epithelial down-growths which evidence the commencement of the stage of malignancy. That the changes in the sub-epithelial tissues in some way play the part of a condition precedent to the exercise of malignant characters by the epithelial cells overlying them appears to be established, though the "how" remains a secret. But once malignant attributes are assumed, the subsequent spread of the disease, whether this be effected by an advanced extension of the primary growth, or by metastases, appears to be independent of pre-existing alterations in the neighbouring connective tissue. Here the invaded tissues play an entirely passive part. There is no cellular infiltration, and the histological evidence is strongly against the existence of any qualities in a cancer cell capable of exciting an inflammatory tissue-reaction. This difference in the behaviour of the connective tissues appears to supply an insuperable argument in favour of Dr. Bonney's view, that the inflammatory processes constantly found in areas of primary carcinoma are in truth pre-carcinomatous, and not secondary.

It is a singular circumstance that, although the inflammations leading up to the pre-carcinomatous stage vary greatly in type, this stage exhibits the same cytological and histological picture whenever it occurs. The predominant items are, as has been said, destruction of normal elastic fibres, and an accumulation of plasma-cells and lymphocytes, to the exclusion of such common types of inflammatory cells as the polymorphonuclear and large hyaline. Over this altered tissue, lies an epithelial layer, which, though at this stage generally thinner than the normal, yet presently becomes endowed with a capacity for proliferation intrinsically, to all appearance, illimitable.

It is a well-established fact that squamous-celled carcinoma, in all respects identical with the common types of this growth, but of somewhat abnormally reduced malignancy, is occasionally a complication of

$x$-ray dermatitis; and this disease, owing to its accessibility and the long duration of its innocent phase, offers peculiar advantages for the study of the histological changes preceding the establishment of malignancy. Mr. Cecil Rowntree, in the same issue of the "Archives of the Middlesex Hospital" gives a good account of these changes. The chief points in the clinical course of the malady, as given by Dr. Hall Edwards, who has himself suffered severely, are as follows: "The disease, so far as the hands are concerned, makes its first appearance as an erythema round the base of the nails. The skin becomes uniformly red; and, later, small warty growths appear, which gradually increase in size and number, the skin at the same time becoming dry and wrinkled. . . . The bases of some of the large warts become inflamed, and the thickened mass comes away, leaving an ulcer which takes months to heal. . . . It is ulcers of this type which occasionally entirely refuse to heal, become gradually larger, and assume malignant characters." Mr. Rowntree was able to obtain material illustrating the structural features associated with the warty condition above described, at a stage before ulceration had appeared. The patient was a man who, after constant daily exposures to the $x$-rays for three years, developed dermatitis of the hands, and began to suffer severe pain in a wart on his right index finger. This wart slowly grew, and three years later was removed on account of the pain it gave. Microscopically the wart was a simple papilloma. There was no actual invasion of the corium, but the deeper layers of epithelial cells were irregularly arranged. The underlying elastic tissue had disappeared, its place being taken by an infiltration of lymphocytes and plasma cells—a type of tissue, therefore, corresponding exactly with that described by Dr. Bonney as the immediate antecedent of malignant epithelial downgrowth. Such researches as these, combined with what we know of carcinoma from the clinical standpoint, make it quite clear that there is no specificity about the kind of irritation which leads up to carcinoma. Mechanical, physical, and bio-chemical agents seem to be equally endowed with the sinister quality of preparing the tissues for carcinomatous invasion, and the final stage in these preparations is a distinctive and unvarying modification of the subepithelial structures.

